# Differences in Dietary Habits, Physical Exercise, and Quality of Life between Male and Female Patients with Overweight

**DOI:** 10.3390/ijerph182111255

**Published:** 2021-10-26

**Authors:** Carmen Herrera-Espiñeira, Manuel López-Morales, María Milagrosa Olmedo-Alguacil, María del Carmen Martínez-Cirre, Antonia Lozano-Sánchez, Inmaculada Cobo-Porcel, Manuela Expósito-Ruíz

**Affiliations:** 1 Faculty of Health Sciences, University of Granada, 18012 Granada, Spain; malomorales@ugr.es; 2 National Network of Research in Health Departments and Chronic Diseases (REDISSEC), 18012 Granada, Spain; 3 Instituto de Investigación Biosanitaria ibs. GRANADA, 18012 Granada, Spain; icoboporcel@gmail.com; 4 Granada-Metropolitan Health District, 18012 Granada, Spain; 5 Faculty of Health Sciences, University of Granada, 51001 Ceuta, Spain; milaolmedo@ugr.es; 6 Clinical Documentation Unit, Virgen de las Nieves University Hospital of Granada, 18014 Granada, Spain; carmen.martinez.cirre.sspa@juntadeandalucia.es; 7 Department of Internal Medicine, Baza Hospital, 18800 Baza, Spain; antonia.lozano.sanchez.sspa@juntadeandalucia.es; 8 Unit of Biostatistics, Department of Statistics, School of Medicine, University of Granada, 18012 Granada, Spain; mexpositoruiz@ugr.es

**Keywords:** women, men, overweight, quality of life, exercise, food habits, patients, internal medicine

## Abstract

Overweight can be an additional problem in patients admitted to hospital. Objective: To analyze gender differences in pre-admission dietary habits and physical exercise and in HRQoL at hospital discharge among hospitalized adults with overweight. Methods: Cross-sectional study in non-diabetic patients enrolled in a clinical trial with body mass index (BMI) ≥ 25 Kg/m^2^ at admission. Bivariate analyses used Pearson’s chi-square test and Fisher’s exact test for qualitative variables and the Mann–Whitney test for numerical variables. Results: The study included 148 males and 127 females. At admission, women had higher BMI (*p* = 0.016) than men and a larger percentage consumed drugs for depression (*p* = 0.030) and anxiety (*p* = 0.049), and followed a religion-based diet (*p* = 0.022). Pre-admission, women had healthier habits related to dietary caloric intake (*p* = 0.009) and greater adherence to recommendations for a healthy diet (*p* = 0.001). At discharge, women described worse self-perceived health (*p* = 0.044) and greater pain/discomfort (*p* = 0.004) in comparison to men. Conclusions: Pre-admission, women had better habits related to a healthy diet and did not differ from men in habits related to physical exercise but had a higher BMI. At discharge, women reported worse self-perceived health and greater pain/discomfort. These differences should be considered for the adequate clinical management of patients with overweight.

## 1. Introduction

The World Health Organization (WHO) [1] has described the main cause of obesity and overweight as an energy imbalance between calories consumed and expended. The incidence of obesity has risen worldwide due to increases in the intake of high-calorie food and in sedentariness, among other reasons. Complex interactions among biological, behavioral, social, and environmental factors are implicated in obesity [2]. The BMI is described as a useful but approximate measure of overweight/obesity, given that it may not correspond to the same degree of adiposity in different individuals. According to the WHO, overweight (BMI ≥ 25 kg/m^2^) is more frequent among women than men worldwide [1] but is more prevalent among men in the European Union (EU) [3].

Multiple factors influence the relationship of females and males with diet and physical exercise. For instance, many women spend a large part of their day devoted to food preparation, implying a closer relationship with the diet [4], and obesity appears to be more socially stigmatized in females [5]. It has also been found that girls show greater dissatisfaction with their body image in response to idealized images in comparison to boys [6]. So-called “emotional eating” and depressive symptoms affect the diet in various ways [7], and a bidirectional risk between depression and obesity has been described as greater for women [8]. In addition, physical activity more frequently complies with recommendations in men than in women [9], who differ in their attitudes towards its practice [10]. In Spain, sedentarism and obesity are more frequent in less privileged social classes, especially among women [11].

An elevated BMI is a major risk factor for cardiovascular diseases (mainly heart disease and stroke), diabetes, musculoskeletal disorders, and some cancers, and the risk is greater with higher BMI [1]. It has been reported that health-related quality of life (HRQoL) is also lower with higher BMI [12]. Overweight/obesity has been associated with worse physical functioning and more pain in females than in males [13], and the increased pain observed with higher BMI is reported to be worse with higher age and to be greater in women than in men [14]. In addition, a survey of the Spanish general population reported a higher mean EQ-5D-5L visual analog score (VAS), i.e., better self-perceived health, in men than in women [15].

It does not appear to be known whether the BMI and overweight-related habits reported for men and women in the general population [1,3,9,14,15] are different in patients with overweight whose severe state of health has led to their hospitalization, and no data could be traced on the HRQoL of these patients at hospital discharge. The specific objective was to explore differences between overweight women and men in their pre-admission dietary habits and physical exercise and in their HRQoL at hospital discharge.

## 2. Material and Methods

A cross-sectional study was conducted in patients with overweight admitted to Internal Medicine (IM) departments and enrolled in a multi-center clinical trial of education on healthy diet and physical exercise.

### 2.1. Study Population

The study included adults admitted to the IM departments of Spanish hospitals Virgen de las Nieves University Hospital, Granada; Baza Regional Hospital; Motril Regional Hospital; and University Hospital of Ceuta who met eligibility criteria for the clinical trial and gave their written informed consent.

### 2.2. Inclusion and Exclusion Criteria

Inclusion criteria were: age ≥ 18 years and BMI ≥ 25 kg/m^2^ at admission to the participating hospitals between February 2018 and February 2020. Exclusion criteria were: a diagnosis of diabetes before admission or during hospital stay, in order to avoid interference with or influence from the multidisciplinary plan for this disease implemented by the regional health ministry [16]; cognitive or physical status hampering questionnaire completion or fulfillment of the clinical trial recommendations for physical exercise and diet, even with the assistance of a caregiver; pre-admission weight-loss diet controlled by nutritionist/endocrinologist; and the receipt of major surgery during the hospital stay.

### 2.3. Evaluation Protocol and Variables Considered

Patients meeting study eligibility criteria were enrolled at hospital admission, when a specifically trained nurse at each center recorded their sex and age, measured their weight and height (barefoot and in pajamas), gathered data from each patient on schooling level (no schooling or primary, secondary, or higher education), work situation (unemployed, actively employed, student, retired, or with permanent disability), cohabitation (living alone or accompanied), smoking (yes/no), pre-admission religion-based diet, pre-admission medication for anxiety or depression (anxiolytics and antidepressants), diagnoses at admission and administered the following questionnaires: Spanish adaptation [17] of the Charlson combined comorbidity index [18] and the Pardo questionnaire [19] on pre-admission dietary and physical exercise habits.

At hospital discharge, a specifically trained nurse recorded the days of hospital stay and administered the validated Spanish adaptation [20] of the EQ-5D-5L quality of life questionnaire, including the VAS [21].

The diagnoses at admission were grouped according to the 10th revision of the International Classification of Diseases (ICD 10) [22].

### 2.4. Measurement Instruments

The Charlson combined comorbidity index [17] estimates the risk of death as a function of comorbidities. It generates a combined global index of age and comorbidity, including 19 diseases with scores between 1 and 6 and adding 1 for each decade past the age of 40 years.

The Pardo Questionnaire [19] was used to quantify pre-admission life habits related to overweight and obesity. It includes 22 questions grouped in five dimensions/factors: (1) energy or calorie content of diet (CC dimension), with 8 items; (2) habitual or systematic practice of physical exercise (PE dimension), with 3 items; (3) healthy diet (HD dimension), with 6 items; (4) alcohol consumption (AC dimension), with 2 items; and (5) food intake for psychological wellbeing (PW dimension), with 2 items. The five response options: never, rarely, sometimes, often, and always are available for all items except for the two items “consume drinks with low alcohol content (beer, wine)” and “consume drinks with high alcohol content (liqueurs, gin, whisky)”, to which the responses are never, once a month, once a week, several times a week, or every day. Item scores range from 1 to 5 and the score for each dimension is obtained by dividing the total score for the items in each dimension by the number of items it contains. Questions with reverse scores were transformed before calculating the dimension, so that higher scores indicated superior habits/wellbeing status in all dimensions (5 points to 1 point and vice versa, 2 points to 4 points and vice versa).

The EQ-5D-5L [20] measures self-perceived health on the day of its completion and comprises: a descriptive section with five dimensions (mobility, personal care, daily activities, pain/discomfort, and anxiety/depression), each with five response options (higher score = worse status); and a VAS for self-perceived health status (0 = worst to 100 = best imaginable).

In accordance with the protocol of the clinical trial in which the present subjects participated, the Pardo questionnaire was administered at hospital admission to evaluate previous habits related to their overweight condition, and the EQ-5D-5L was administered at discharge to determine the HRQoL with which they returned home.

The study complied with EU regulations (2016/679) and Spanish legislation (3/2018) on personal data protection and digital rights and was conducted in accordance with the 2013 revision of the Declaration of Helsinki (https://www.wma.net/what-we-do/medical-ethics/declaration-of-helsinki/; accessed on 13 July 2017). All subjects gave their informed consent to participate in the study, which was approved by the clinical research ethics committees of Andalusia and the four participating hospitals.

### 2.5. Statistical Analysis

In a descriptive analysis, frequencies and percentages were calculated for qualitative variables and medians with interquartile range (*IQR* [*P25–P75*]) for numerical variables because of their non-normal distribution (Kolmogorov–Smirnov test). Bivariate analyses were performed, applying Pearson’s chi-square test and Fisher’s exact test for qualitative variables and the Mann–Whitney test for numerical variables. *p <* 0.05 was considered significant. IBM SPSS 19 software (IBM Corp., Armonk, NY, USA) was used for statistical analyses.

## 3. Results

The study included 275 patients, 148 males (53.6%) and 127 females (46.3%), with a median age of 71.5 years (IQR [58–81]).

It is estimated that around 1000 non-diabetic patients with obesity are annually admitted to the participating Intensive Medicine Departments. Therefore, the study population of 275 patients enrolled during the study period represents 13.75% (275/2.000) of the total number admitted with obesity.

Data on the diagnoses at admission are compiled in Table 1, showing that there was no significant difference in diagnostic groups between men and women (*p* = 0.086). The most frequent diagnoses, in 79% (*n* = 111) of the men and 72.6% (*n* = 90) of the women, were for other diseases of organs and body systems (codes G00–N99), which were grouped together due to the small number of cases available for each code.

There was no gender difference in schooling (*p* = 0.143), cohabitation (*p* = 0.769), smoking status (*p* = 0.276), length of hospital stay (*p* = 0.591), Charlson Comorbidity Index (*p* = 0.858), or age (*p* = 0.091), There were differences in work situation (*p* = 0.032), with men being more frequently active or students, 23.6% (*n* = 34) vs. 11.9% (*n* = 15). The women more frequently followed a religion-based diet, 20.2% (*n* = 22) vs. 9.8% (*n* = 13); *p* = 0.022. They also more frequently consumed drugs for depression, 16.1% (*n* = 20) vs. 7.6% (n11); *p* = 0.030 or anxiety, 15.2% (*n* = 19) vs. 7.6% (*n* = 11); *p* = 0.049, and had a higher median BMI, 31.96 vs. 30.6 Kg/m^2^; *p* = 0.016 (Table 2).

There was no gender difference in EQ-5D-5L dimensions except for pain/discomfort, which was greater in women (*p* = 0.004), and worse median self-perceived health by VAS, 50 vs. 60; *p* = 0.044 (Table 3).

According to the Pardo questionnaire, women had better habits related to dietary caloric content (CC dimension, median 2.25 vs. 2; *p* = 0.009), greater concern for a healthy diet (HD dimension, median 3.67 vs. 3.33; *p* = 0.001), but worse habits related to alcohol consumption (AC dimension, median 3 vs. 3.5; *p* < 0.001). There were no differences in habits related to physical exercise (PE dimension, median 1.33 in both; *p* = 0.432) or food intake for psychological wellbeing (PW dimension, median 4 vs. 4.33; *p* = 0.493) (Table 4).

## 4. Discussion

This study of overweight adults admitted to IM departments in Spain observed differences between women and men. A main finding was that the pre-admission diet of the women was healthier in relation to its caloric content and adhered more closely to WHO recommendations [23] for healthy eating in comparison to the men, worse habits related to alcohol consumption were reported by the women, although a lower test-retest coefficient (0.85) was obtained for this dimension than for the others in the validation study, with a reliability coefficient below 0.75 [19]. This finding should also be considered with caution because of a possible reluctance to admit this behavior, especially in the presence of caregivers, who accompanied many of the older patients. The daily intake of fruit and vegetables is known to be more frequent among women than men in Europe [3] and to be inversely related to the BMI [24]. A study conducted in 23 countries [25] attributed the food choices of women in part to their greater involvement in weight control and stronger interest in the benefits of a healthy diet. In addition, the consumption of alcohol, which is positively related to a higher BMI, carries a greater likelihood of overweight/obesity in men than in women [26]. However, the differences in alcohol consumption between the sexes are decreasing [27,28].

The women more frequently followed a religious-based diet in comparison to the men. However, few data are available on the relationship of this practice with obesity/overweight status. A worldwide study by the Pew Research Center [29] observed that women are in general more committed to religions and their rules in comparison to men, for a variety of possible reasons.

No significant gender difference was found in habits related to the practice of physical activity unrelated to work, whereas this has been described as more frequent among males than females in the general population of the EU [3]. However, it should be borne in mind that the presence of disease or comorbidities can influence the capacity of individuals to perform physical exercise.

Despite their healthier dietary habits, the BMI of the women was higher than that of the men, unlike in the general population of EU countries [3]. However, other studies in hospitalized patients have also described a higher percentage of females than males with overweight or obesity [30,31,32].

Significantly worse pain/discomfort (EQ-5D-5L score) was reported by the women than by the men in the present study, as previously reported [13]. Pain, known to be related to the BMI and age, especially among women [14], can limit mobility [33,34], and a bidirectional risk between weight and mobility impairment can increase over time [35]. This may generate a vicious circle that worsens the situation of patients after hospital discharge and may affect women more strongly, given the present findings of higher BMI and greater pain in the female versus male patients. Reasons for the higher perception of pain by women have not been fully elucidated, although some gender differences in endogenous pain-inhibiting systems, cognitive/social factors, and personal histories may play a role [36].

Anxiety and depression are associated with a worse HRQoL [31], and more women than men received medication for these conditions in the present study. Some HRQoL aspects appear to mediate in the relationship between BMI and depression in females but not in males [8,13]. The relationship between emotions and food is well documented [37,38], and binge eating disorder [39] and negative states of mind have been associated with obesity [40].

The median VAS-measured HRQoL of our participants was worse in the women (50 in females vs. 60 in males), similar to observations in a survey of the Spanish general population [15]. However, the men and women in the present study did not differ in the anxiety/depression dimension of the EQ-5D-5L, although this instrument does not discriminate between these two mood disorders. Furthermore, there was no significant difference between men and women in Charlson combined comorbidity index score or in diagnostic groups at admission. Among other proposed reasons for a gender difference in HRQoL is the later hospital admission of women in a worse clinical condition, as found by a study on hip or knee arthroplasty [41]. Karlson et al. [42] suggested that one reason for this delay may be that women are more concerned about increasing the burden on others.

A difference was observed in work activity outside the home, which was reported by a lower percentage of the women in our study, consistent with previous findings in EU countries. There was no gender difference in smoking habit, which was found to be more frequent in males in the EU [3]. However, patients with overweight and comorbidities are likely to receive constant warnings from health care professionals to quit the habit, which may have reduced this tendency in our patients.

Our findings underscore the need for further in-depth research into the causes of gender-based health disparities and for the implementation of clinical approaches, secondary prevention measures, and weight reduction programs that take account of differences between the sexes.

Study limitations: The participants were enrolled over two years, because the originally planned one-year period yielded an inadequate sample of patients, largely due to the greater-than-expected percentage of patients with diabetes or unable to complete the questionnaire because of cognitive impairment. In addition, a different nurse was responsible for data gathering in each center, although all were trained for this purpose by the same researcher, reducing possible variability. Finally, it proved necessary to group together the wide range of diagnoses recorded at admission. Larger patient samples are required in order to analyze the influence of specific diagnoses on differences between the sexes. Further research is also needed to explore the possible bias in alcohol consumption reporting by these patients.

The sample size was not calculated for this cross-sectional study, which was a secondary objective of a clinical trial and limited to analyzing data gathered from patients randomly selected for the trial in accordance with its eligibility criteria.

## 5. Conclusions

Before hospital admission, the women had healthier dietary habits and did not differ from the men in physical exercise practice or comorbidity index score; nevertheless, they had a higher BMI. At discharge, the women reported worse self-perceived health and greater pain/discomfort in comparison to the men. There is a need to promote healthy habits in patients with overweight and to take special account of the pain experienced by the women in order to improve the clinical management of this population.

## Figures and Tables

**Table 1:** Distribution of diagnoses at patient admission.

DIAGNOSIS GROUPING (ICD 10)	Men *n* (%)	Women *n* (%)	Total
G1 Certain infectious and parasitic diseases (A00–B99)	3 (2.1)	4 (3.2)	7 (2.6)
G2 Neoplasms (C00–D49)	3 (2.1)	5 (4.0)	8 (3.0)
G3 Diseases of the blood and blood-forming organs and certain disorders involving the immune mechanism (D50–89)	2 (1.4)	2 (1.6)	4 (1.5)
G4 Endocrine, nutritional, and metabolic diseases (E00–E89)	2 (1.4)	3 (2.4)	5 (1.9)
G6 Other diseases of organs and body systems (G00–N99)	113 (79.0)	90 (72.6)	203 (76.0)
G9 Various groups (R00–Z99)	20 (14.0)	20 (16.1)	40 (15.0)
Total	143 (100)	124 (100)	267 (100)

*p =* 0.086. ICD 10: 10th revision of the International Classification of Diseases. G6 includes diseases with codes G00–G99, H00–H59, I00–I99, J00–J99, K00–K95, L00–L99, and M00–M99.

**Table 2:** Comparison between sexes in sociodemographic data, clinical variables.

Variables	Men	Women	*p*
	148 (54%)	127 (46%)	
	*n* (%)	*n* (%)	
**Schooling**			0.143
None	38 (26.2)	44 (34.9)	
Primary	68 (46.9)	59 (46.8)	
Secondary/university	39 (26.9)	23 (18.3)	
**Work situation**			0.032
Unemployed	11 (7.6)	15 (11.9)	
Actively employed/student	34 (23.6)	15 (11.9)	
Retired/permanent disability	99 (68.8)	96 (76.2)	
**Cohabitation** (lives alone)	19 (13.2)	15 (12)	0.769
**Smoker** (yes)	20 (13.7)	12 (9.4)	0.276
**Practices religion-based diet**	13 (9.8)	22 (20.2)	0.022
**Takes anti-depression drug**	11 (7.6)	20 (16.1)	0.03
**Takes anti-anxiety drug**	11 (7.6)	19 (15.2)	0.049
**Days of stay** ^a^	8 [6–10.25]	8 [6–10]	0.591
**Charlson index** ^a^	3 [1–4]	3 [1–5]	0.858
**BMI** ^a^	30.6 [28.65–33.25]	31.96 [29.3–37.9]	0.016
**Age** ^a^	70 [55.5–78.5]	72 [58–83]	0.091

^a^ Median [percentile 25–percentile 75]. BMI = body mass index.

**Table 3:** Results of EQ-5D-5L questionnaire at discharge.

Dimensions with Response Options	Men 148 (54%)	Women 127 (46%)	Total 276 (100%)	*p*
	*n* (%)	*n* (%)	*n* (%)	
**Mobility**				0.358
No problems to walk	60 (40.3)	51 (40.5)	111 (40.4)	
Mild problems to walk	35 (23.5)	19 (15.1)	54 (19.6)	
Moderate problems to walk	26 (17.4)	29 (23.0)	55 (20.0)	
Severe problems to walk	18 (12.1)	20 (15.9)	38 (13.8)	
Cannot walk	10 (6.7)	7 (5.6)	17 (6.2)	
**Personal care**				0.567
No problems to wash or get dressed	91 (61.1)	66 (52.0)	157 (56.9)	
Mild problems to wash or get dressed	18 (12.1)	20 (15.7)	38 (13.8)	
Moderate problems to wash or get dressed	19 (12.8)	19 (15.0)	38 (13.8)	
Severe problems to wash or get dressed	13 (8.7)	16 (12.6)	29 (10.5)	
Cannot wash or get dressed	8 (5.4)	6 (4.7)	14 (5.1)	
**Daily activities**				0.192
No problems to perform daily activities	84 (57.1)	55 (43.3)	139 (50.7)	
Mild problems to perform daily activities	18 (12.2)	26 (20.5)	44 (16.1)	
Moderate problems to perform daily activities	18 (12.2)	18 (14.2)	36 (13.1)	
Severe problems to perform daily activities	19 (12.9)	20 (15.7)	39 (14.2)	
Cannot perform daily activities	8 (5.4)	8 (6.3)	16 (5.8)	
**Pain/discomfort**				0.004
No pain/discomfort	73 (49.3)	38 (29.9)	111 (40.4)	
Mild pain/discomfort	46 (31.1)	42 (33.1)	88 (32.0)	
Moderate pain/discomfort	21 (14.2)	34 (26.8)	55 (20.0)	
Strong pain/discomfort	7 (4.7)	13 (10.2)	20 (7.3)	
Extreme pain/discomfort	1 (0.7)	0 (0.0)	1 (0.4)	
**Anxiety/depression**				0.619
No anxiety or depression	79 (53.0)	59 (46.8)	138 (50.2)	
Mildly anxious or depressed	47 (31.5)	42 (33.3)	89 (32.4)	
Moderately anxious or depressed	15 (10.1)	14 (11.1)	29 (10.5)	
Very anxious or depressed	6 (4.0)	10 (7.9)	16 (5.8)	
Extremely anxious or depressed	2 (1.3)	1 (0.8)	3 (1.1)	
**Q-5D-5L VAS score ^a^**	60 [50–70]	50 [41.25–70]		0.044

^a^ Median [percentile 25-percentile 75].

**Table 4:** Results of PARDO questionnaire dimensions on life habits related to overweight before hospital admission.

Pardo Questionnaire	Men, *n* = 148 (54%) Women, *n* = 127 (46%)		*p*-Value
Dimensions	*Median [P25–P75]* Dimension score		
Calorie content of diet	2 [1.75–2.37]	2.25 [1.81–2.62]	0.009
Food intake for psychological wellbeing	4.33 [3.67–5]	4 [3.33–5]	0.493
Regular practice of physical exercise	1.33 [1–2.33]	1.33 [1–2]	0.432
Concern for healthy diet	3.33 [3–3.83]	3.67 [3.33–4]	0.001
Alcohol consumption	3.5 [3–4]	3 [3–3.5]	<0.001

*Median [P25–P75]:* median [percentile 25–percentile 75].

## Data Availability

The underlying data are available on request.
